# All-cause and Cardiovascular mortality among ethnic German immigrants from the Former Soviet Union: a cohort study

**DOI:** 10.1186/1471-2458-6-16

**Published:** 2006-01-26

**Authors:** Ulrich Ronellenfitsch, Catherine Kyobutungi, Heiko Becher, Oliver Razum

**Affiliations:** 1 Department of Tropical Hygiene and Public Health, University Hospital of Heidelberg, INF 324, 69120 Heidelberg, Germany; 2 Department of Epidemiology and International Public Health, School of Public Health, University of Bielefeld, P.O. Box 10 01 31, 33501 Bielefeld, Germany

## Abstract

**Background:**

Migration is a phenomenon of particular Public Health importance. Since 1990, almost 2 million ethnic Germans (*Aussiedler*) have migrated from the former Soviet Union (FSU) to Germany. This study compares their overall and cardiovascular disease (CVD) mortality to that of Germany's general population. Because of high overall and CVD mortality in the FSU and low socio-economic status of *Aussiedler *in Germany, we hypothesize that their mortality is higher.

**Methods:**

We conducted a retrospective cohort study for 1990–2002 with data of 34,393 *Aussiedler*. We assessed vital status at population registries and causes of death at the state statistical office. We calculated standardized mortality ratios (SMRs) for the whole cohort and substrata of covariables such as age, sex and family size. To assess multivariate effects, we used Poisson regression.

**Results:**

1657 cohort members died before December 31, 2002, and 680 deaths (41.03%) were due to CVD. The SMR for the whole cohort was 0.85 (95%-CI 0.81–0.89) for all causes of death and 0.79 (95%-CI 0.73–0.85) for CVD. SMRs were higher than one for younger *Aussiedler *and lower for older ones. There was no clear effect of duration of stay on SMRs. For 1990–93, SMRs were significantly lower than in subsequent years. In families comprising at least five members upon arrival in Germany, SMRs were significantly lower than in smaller families.

**Conclusion:**

In contrast to our hypothesis on migrants' health, overall and CVD mortality among *Aussiedler *is lower than in Germany's general population. Possible explanations are a substantially better health status of *Aussiedler *in the FSU as compared to the local average, a higher perceived socio-economic status of *Aussiedler *in Germany, or selection effects. SMR differences between substrata need further exploration, and risk factor data are needed.

## Background

Migration is a phenomenon reshaping society structure in numerous countries [1]. It is of particular Public Health importance because the health status of migrants frequently differs from that of the general population of the country these people come to, the "host country", as well as from that of the population of the country they come from, the "country of origin" [2,3].

Since the early 1990s, almost 2 million ethnic German "resettlers" (*Aussiedler*) have migrated from the FSU to Germany [4]. Being descendants of people who left what is now Germany to Russia centuries ago, *Aussiedler *suffered strong discrimination during and after the two World Wars, but partially managed to keep their own ethnic identity. In the aftermath of the collapse of the Soviet Union, they began to migrate to Germany in large numbers. German legislation favours these migrants through social benefits and by giving them German nationality upon arrival in the country. This distinguishes them from other migrants, such as labour migrants, who receive much less support from the state [5].

So far, there are no data on overall and cause-specific mortality in this population group. The present study aims at filling this gap by providing data on overall mortality and mortality from CVD, the most frequent cause of death in the industrialized world [6], for *Aussiedler *from the FSU, and by comparing it to that of Germany's general population. In a more general context, a hypothesis on the health status of migrants is developed and tested in this population.

There are numerous studies from different countries showing that the health status and mortality of migrants differ from that of the host country's general population. Often, migrants have a lower mortality which gradually converges towards mortality levels prevailing in the host country with increasing duration of stay and over generations. This pattern is particularly common for CVD and other chronic conditions [2,3,7]. A closer look, however, reveals that such an initial mortality advantage almost exclusively occurs in migrants from populations where mortality from these diseases is lower than in the host country. One example can be found in the United States, where this is the case for migrants from Latin America and Asian-Pacific islands, both regions with lower chronic disease mortality [7]. Conversely, migrants from a country where chronic disease mortality is higher than that in the host country have an initially higher mortality. This, for example is the case for migrants from Finland to Sweden [8]. Similar data are reported from Canada. There, migrants from Scandinavian countries, where CVD mortality is high, had an increased risk of dying from CVD compared to those from regions with a lower CVD mortality like Latin America and Asia [9]. In general, overall and CVD mortality in migrants seems to converge from an initial level similar to that in the country of origin towards the level in the host country [3]. It can be claimed that this pattern is determined by factors from three broad categories, namely factors in the country of origin, factors in the host country, and migration-specific factors.

For *Aussiedler *from the FSU, these factors potentially determine overall and CVD mortality as follows. First, in the countries of origin, the FSU, overall mortality is substantially higher than in Western Europe, which is to a large extent a consequence of extraordinarily high CVD mortality. In fact, FSU countries have the highest CVD mortality worldwide with mortality rates being about three times that of Western European countries. For example, the age-standardized CVD mortality rate in the FSU countries in 2000 was 772.05 per 100,000, whereas in the European Union it was 249.89 per 100,000. Respective figures for all-cause mortality were 1371.74 and 665.91 [6]. Thus, the largest part of the East-West mortality gap is attributable to deaths from CVD [10]. The reasons for this phenomenon are not fully understood, but most probably the driving force is a combination of various risk factors like smoking, nutritional deficiencies and binge drinking [10,11]. Mortality in the FSU seems to differ between certain population subgroups with different ethnic and religious backgrounds, and at least some of these observed differences are due to concomitant disparities in socioeconomic characteristics [12,13]. For *Aussiedler *in the FSU, there are no specific mortality data but there is some information on SES. The few existing studies in the countries of origin showed that economically *Aussiedler *fared somewhat better than the general population. There were no differences with regard to employment or education, but the importance of family and religion seemed to be higher among *Aussiedler *than the general population [14,15]. In summary, there is little evidence for differences in SES which could explain a substantially different risk factor profile. Because the gap in mortality averages between the two countries is very big, overall and CVD mortality among *Aussiedler *is probably higher than in Germany's general population *before *migration to Germany. Due to a presumed "time lag", i.e. a long latency between exposure and onset of disease, for some risk factors [16,17], CVD mortality should remain high, or even increase, compared to that of the general population of Germany, *after *the *Aussiedler's *arrival in Germany.

Second, studies have shown that *Aussiedler *from the FSU in Germany, in spite of their privileged status compared to other migrants, have a lower SES than the country's general population. This has manifold reasons among which there might be the mere fact of being a migrant, i.e. a person who has to adapt to a new environment and society [18-20]. Low SES is independently associated with high overall and CVD mortality in many populations around the world [16,21,22]. Therefore, this observed socio-economic disadvantage is another potential reason for a higher overall and CVD mortality of *Aussiedler *from the FSU as compared to Germany's general population.

Third, factors related to migration itself might have an effect on the subsequent mortality of migrants. In many instances, migrants seem to be a selected sample of their population of origin. Such selection can be based on health status, but there are arguments for either direction of this effect. Possibly, particularly healthy people decide to migrate, which is referred to as the "healthy migrant effect", or more sick people do so because they may be hoping for better treatment in the country of destination [3]. It is important to notice that such selection only occurs compared to the population of origin, and not compared to the host country's population [3,23]. In the case of *Aussiedler *from the FSU, it is hard to quantify how much health selection took place during migration. Since the large majority of *Aussiedler *in the FSU has now migrated to Germany [24], the overall effect of selection is probably rather small. Another potential migration-specific factor with beneficial influence on health status is the formation of a strong ethnic identity within families and social networks in the host country [25], and indeed there is some evidence for the formation of such a specific identity among *Aussiedler *from the FSU in Germany [26,27].

In summary, we expected that *Aussiedler *from the FSU in Germany have a higher overall and CVD mortality than Germany's general population. Migration-specific factors might have an alleviating effect on their mortality.

To test this hypothesis, and to provide data for resource allocation and health planning in Germany as well as in other countries which experience in-migration at large scale, we conducted a retrospective cohort study among a representative sample of *Aussiedler *from the FSU. We investigated possible mortality differentials between subgroups of this population and assessed the effect of covariables available in this study.

## Methods

We conducted a retrospective cohort study with the aim of recruiting a study population as representative as possible of the totality of *Aussiedler *from the FSU who came to Germany. Because the focus of our study was on adult mortality, we confined the study population to persons aged at least 15 years upon arrival in Germany. The sampling and follow-up procedures have been described in detail elsewhere [28]. In short, we obtained a dataset containing individual data of all *Aussiedler *from the FSU (n = 281,356) who had come to North-Rhine Westphalia, Germany's most populous federal state, between 1990 and 2001. The final cohort for analysis consisted of all those for whom an automated record linkage with the population registry of their first residence was possible (n = 34,394). Assignment of *Aussiedler *to the different federal states and municipalities within Germany is done according to a quota considering the population size of the respective state and municipality. For example, North-Rhine Westphalia, where about 21% of the German population live, receives 20.8% of all *Aussiedler *coming to Germany [24]. Arriving *Aussiedler *can express a preference for a state but respective assignment is by no means guaranteed. This can be considered as a quasi-random procedure and therefore we assume that our selection technique delivers a random sample of all *Aussiedler *who arrived in Germany during the study period [5,28].

For the cohort, we assessed vital status, i.e. if a person is either alive or deceased, at the end-of-follow-up, December 31, 2002. All observations were censored at that date. We calculated person time with the date of arrival in Germany as start and either the predefined end of follow-up, the date of death or the date of last known vital status, as end of the observation period. Person time was allocated to strata of approximately equal width formed by different values of the covariables available in our dataset (sex, age, study period, year of arrival in Germany, duration of stay in Germany, family size at arrival and population size of first residence). The definition of strata is given in table 1. Stratification according to family size upon arrival in Germany was done in the categories "arrived alone" – "arrived within a family of two to four members" – "arrived within a larger family". The rationale for this stratification is based on the finding that in Germany, singles, i.e. people without partner or spouse as well as divorced or widowed persons, have a significantly higher mortality than those living with a partner [29], and on the fact that for *Aussiedler *large family networks appear to play a crucial role in everyday life [25]. As cut-off point for the stratification of population size of the city of arrival, we used a population size of 100,000.

For deceased participants, we ascertained cause of death coded according to ICD in an anonymous way at the state statistical office of North-Rhine Westphalia [30]. In the rare case of non-availability of cause of death (4.2% of requested cases), we retrieved death certificates at local health authorities. The listed causes were subsequently coded according to ICD by professional coders at the Saarland cancer registry. We obtained all causes of death according to ICD9 for deaths which occurred before December 31, 1997, and according to ICD10 for deaths thereafter. We defined CVD deaths as deaths with an ICD9 code between 390 and 459 or an ICD10 code between I00 and I99 [31,32].

We calculated SMRs for all causes and CVD with observed and expected deaths for each calendar year, sex, and five-year age group. The number of expected deaths was based on publicly available mortality data and mid-year population figures for the same five-year age groups of the entire resident population of Germany [33]. Regional mortality data for the state of North-Rhine Westphalia are available, but figures are highly similar to those for the whole of Germany [33,34]. Since our aim was to draw conclusions on a country level, we decided to use data of Germany's entire resident population to calculate expected deaths.

We performed SMR calculation in the whole cohort as well as in the different substrata. Since the percentage of cases with missing information on the cause of death was negligible, we did not apply a correction technique [35] for CVD SMRs. We calculated 95 % confidence intervals for SMRs with approximate methods when the number of observed cases was 50 or larger and with exact methods if it was smaller [36]. In addition, to compare secular trends of CVD mortality in the two populations, we used direct standardisation. As standard population, we employed a truncated form (only age groups above 15 years) of the European Standard Population [37]. We calculated corresponding 95 % confidence intervals from the standard errors of the age-specific rates under the assumption that their covariances are zero [36].

To assess the simultaneous influence of the several measured covariables, we used Poisson regression to obtain univariate and multivariate-adjusted estimates for the effect of covariables on SMRs [36]. Here, the SMR is modelled in dependence of the values of the covariables by assuming that the number of events in a given stratum is Poisson-distributed and by using the natural logarithm of expected cases for each stratum as an offset term. The strata are formed by cross-classification of all covariables as given in table 1. From the coefficients we calculated rate ratios. Corresponding 95 % confidence intervals were obtained with maximum likelihood methods. We used SAS version 8.02 [38] for all statistical analyses.

## Results

### Descriptive statistics

Our final cohort consisted of 34,393 *Aussiedler *from the FSU. The characteristics of cohort members at the start of the observation period, i.e. upon their arrival in Germany, as well as the number of accrued person years in the different strata are shown in comparison to the totality of *Aussiedler *who migrated from the FSU to North-Rhine Westphalia between 1990 and 2001 in table 1. This comparison does not reveal appreciable differences with regard to the available covariables, with the exception of population size of the first residence, which was more frequently above 100,000 in the cohort.

Of the 34,393 cohort members, 29,512 (85.8%) were still alive at the end of follow-up on December 31, 2002. 1,657 (4.8%) were deceased and 2,916 persons (8.5%) had moved to a municipality where no further follow-up had been performed. Of these 2,916 persons, 188 (0.5%) had moved abroad, all but 10 of them to the FSU, and 120 (0.4%) had moved to an unknown destination. Out of the 1,657 deaths, we were able to obtain the cause for 1628 (98.3%). 680 (41.8%) met the definition of a CVD death. 292 (17.9%) were due to coronary heart disease (ICD9 410–414 / ICD10 I20–I25), 136 (8.4%) due to cerebrovascular accidents (ICD9 415–417 / ICD10 I60–I69), and 252 (15.5%) due to other CVD (all remaining ICD codes for CVD). SMR and Poisson regression analyses for subforms of CVD did not yield appreciably different results from analyses for CVD as a whole, therefore only the latter are presented.

### SMR analysis and directly standardized mortality

Tables 2 and 3 present observed and expected numbers of deaths and SMRs in the whole cohort and substrata for all causes of death and CVD, respectively. SMRs for CVD and all causes of death show a highly similar pattern.

**Figure 1:**
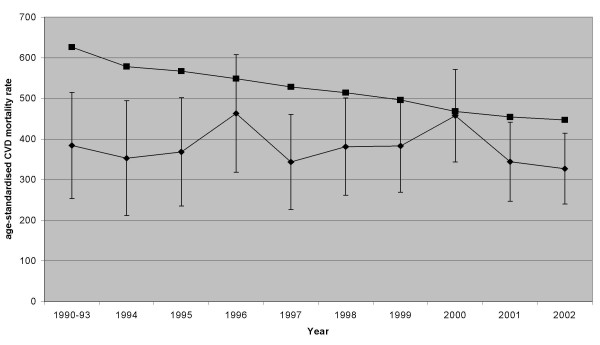
Directly age-standardised CVD mortality rates and 95 % confidence intervals for men in the cohort (◆) and men in the general German population (■). Rates are presented as per 100,000 person years/population.

**Figure 2:**
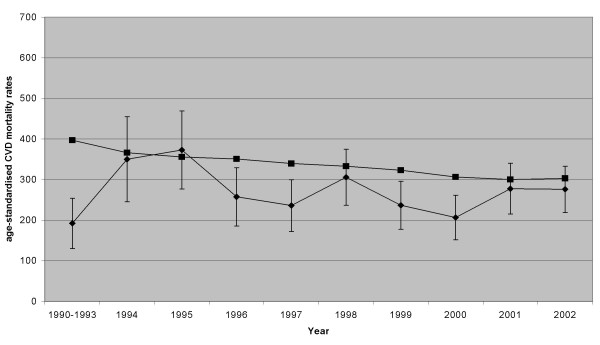
Directly age-standardised CVD mortality rates and 95 % confidence intervals for women in the cohort (◆) and women in the general German population (■). Rates are presented as per 100,000 person years/population.

Overall and CVD mortality in the cohort is significantly lower than in the general population of Germany (overall SMR = 0.85, 95%-CI 0.81–0.89; CVD SMR = 0.79; 95%-CI 0.73–0.85). There are no appreciable differences in SMRs between sexes. There is, however, an age gradient in SMRs, with SMRs being above one in the youngest age group (15–39 years) and significantly below one in the two older age groups. This gradient is more pronounced for CVD than for all causes of death. Increasing duration of stay seems to be associated with a higher SMR for CVD. The values for a stay up to eight years are significantly below one while the confidence interval of the value for a longer stay includes one. In contrast, corresponding SMRs for all causes of death do not show any gradient. Of the other covariables, study period and family size upon arrival show an association with SMRs. For the period 1990–1993, SMRs are significantly lower than for 1998–2002, and this difference is more pronounced for CVD. A comparison of directly standardized CVD mortality rates for different years (figures 1 and 2) reveals that this is due to declining CVD mortality in the general population of Germany and unchanged CVD mortality among *Aussiedler *from the FSU. For persons who arrived in a family comprising five or more members, SMRs are considerably lower than for persons who came in smaller families or as singles. This effect reaches significance in the analysis with all causes of death. Year of arrival and size of the first city of residence do not seem to have an effect on SMRs.

### Poisson regression

Tables 4 and 5 present the results of the Poisson regression models. The rate ratios are measures for the effect of each value of a covariable on the SMR. A rate ratio larger than one means that the SMR in the strata formed by the respective value of the covariable is higher than in the ones formed by the value defined as reference category and vice versa. The univariate models replicate the results from the SMR analysis. Multivariate adjustment does not change most of the rate ratios to an appreciable extent, which shows that there is relatively little confounding by the available covariables. The association between a long duration of stay in Germany and a relatively higher CVD SMR is attenuated after adjusting for other covariables, whereas the positive association between study period and SMR becomes stronger, especially for CVD. The association between large family size and low CVD SMR reaches statistical significance in the multivariate-adjusted model. Results are similar when log (person-years) is used as offset term in Poisson regression (except for age).

## Discussion

To our knowledge, this study is the first of its kind which assesses mortality among *Aussiedler *from the FSU in Germany. In contrast with the postulated hypothesis on the health status of migrants, the findings show that this migrant group has a significantly lower overall and CVD mortality than Germany's general population. CVD is the most important cause of death in this population, and SMR patterns for all causes of death and CVD are highly similar. This suggests that the measured covariables are associated with CVD and overall mortality in a similar way. Stratification and multivariate analyses reveal that there are considerable differences in SMRs between different age groups, calendar years, and between large and small families.

The health of migrants is a complex phenomenon [39], and thus there are no stringent simple explanations for the surprising results. The findings are in contrast to results from studies in the United States, Canada and Sweden which suggest that post-migration CVD and overall mortality in migrants is determined by mortality in the countries of origin [7-9]. Our hypothesis which predicted a higher overall and CVD mortality might have been based on incorrect assumptions. Although overall and CVD mortality are excessively high in the FSU, a finding which is unlikely to be due to non-reliable data [40,41], our assumption that this is also the case for the population of *Aussiedler *in the FSU may have been wrong. Specific data shedding light on this matter do not exist, and the few available studies on SES among *Aussiedler *in the FSU [14] might have not sufficiently been able to measure socio-economic differences that could explain mortality differentials between *Aussiedler *and the population average in the FSU. The special importance of "traditional" family and religious values might determine certain lifestyle-dependent risk factor profiles leading to a lower mortality. A potentially alike pattern can be found among Russian Jews for whom similar values seem to play an important role in everyday life. In Moscow, it was found that they have a mortality advantage compared to the city's general population [12]. This might be one of the reasons for the finding that, contrary to what was expected [42], Jewish migrants from Russia to Israel have a similar to slightly lower CVD and overall mortality when compared to the general population of Israel, whose mortality is much lower than the FSU average [43]. Nevertheless, to fully explain the low mortality found among *Aussiedler *in our study, based on the mortality data for the entire population of the FSU given in the introduction, their overall mortality in the FSU would need to be only 40 % and their CVD mortality only a third of the average in these countries. Intuitively, this seems improbable. However, it is known from many populations around the globe that there are huge disparities in mortality between subgroups. In most countries, women have a mortality rate which is about half that of men. Within the city of Chicago, life expectancy differs with a magnitude of more than 20 years across neighbourhoods [44]. It is possible that such substantial differences exist also between *Aussiedler *in the FSU and the general population, but data to support this hypothesis are not available. Another possibility to verify if there are "ethnicity-specific" rather than "country-of-origin-specific" characteristics determining the mortality of *Aussiedler *would be to compare the mortality of *Aussiedler *from the FSU to that of *Aussiedler *from Poland and Romania in Germany, with whom they share genetic, cultural and environmental characteristics. So far, there are no mortality data for the latter.

The second assumption of our hypothesis, that socio-economic disadvantages of *Aussiedler *lead to higher overall and CVD mortality compared to the general population, might also be erroneous. Most studies measured only "conventional" socio-economic markers such as income, housing and occupational characteristics and did not assess more subjective attributes (e.g. life satisfaction and other psychosocial factors) [18-20]. "Subjective SES" is in some settings more strongly related to health outcomes than objective markers [16]. Thus, higher perceived SES among *Aussiedler *from the FSU might contribute to lower mortality than in Germany's general population. There are hints that life satisfaction among *Aussiedler *is indeed higher than in their German peers [45,46], something which could be favoured by the formation of an own specific "cultural identity"[25] during the transition from life in the FSU to life in Germany, providing strong social cohesion with beneficial effects on health [26,27]. Only further in-depth studies have the potential to test for this speculative assumption.

Strong health selection during migration from the FSU to Germany, in the sense of the frequently postulated "healthy migrant effect" [3], is a rather improbable reason of the observed low mortality. It would need to be very strong in order to explain the observed low SMRs, something which is unlikely because the vast majority of the once existing population of *Aussiedler *in the FSU has migrated to Germany [24]. Selective return migration of sick or socio-economically disadvantaged people with a higher risk of dying, another alleged explanation for observed low overall and CVD mortality in some migrant populations [39,47,48], can be excluded in our study population, of which less than one % moved back to the FSU.

Differences in SMRs between younger and older *Aussiedler *might be due to a particularly poor risk factor profile in young adults, similar to what is observed in the FSU [49,50], or due to age-dependent selection. In older age, where diseases, and especially CVD, often present in a limiting manner, people with prevalent disease might decide against migrating. In younger age, where sudden cardiac deaths without previous limiting disease are more common [6], there might be no such selection.

Observed secular differences in SMRs are much stronger for CVD than for all causes of death. They result mostly from a CVD mortality decline in the German population which *Aussiedler *from the FSU do not experience. The reasons for this decline, which is common to many Western countries are not fully understood, but advances in treatment and prevention seem to be driving forces [51]. Our results imply that *Aussiedler *from the FSU do not benefit from these advances to the same extent as Germany's general population.

The significantly lower SMRs in *Aussiedler *arriving in large families and the much smaller difference in SMRs between singles and families with two to four members are also unexpected. Health effects of family and social ties are as complex a matter as migrants' health [52]. The data available in this study are insufficient to provide convincing explanations. The fact that large family networks seem to play an important role for *Aussiedler *[15] might have helped the ones actually migrating within the texture of a large family to deal better with post-migration stress. Such family resources might be particularly helpful given that by migrating *Aussiedler *undergo a transition from a more "collectivistic" towards a more "individualistic" society. Without specific data, however, this hypothesis is bound to remain speculative. Consequently, to learn more about the observed association, studies focusing on the quantity and quality of inter-personal relationships of migrants, and of *Aussiedler *in particular, are urgently needed.

Due to its size, our study is able to provide reliable data on this population's overall and CVD mortality. Although the sampling scheme was partly guided by pragmatic concerns, a comparison shows that there are no appreciable differences with regard to the measured covariables between cohort members all *Aussiedler *from the FSU. One exception is that *Aussiedler *who had been assigned to larger cities were overrepresented in our cohort. Since we did not find any mortality differences between large and small cities, this should not have biased our results. We thus believe that our cohort constitutes a representative sample of the population under study [28].

We could not further follow up 8.5 % of cohort members after they had moved to another municipality. This would cause an appreciably bias only if the mortality in this subgroup was very different to the main part of the cohort which we think is very unlikely. The German population registry system is known to be not fully accurate [53], but potential mistakes in registration affect data from the cohort and the general population similarly. The same holds true for information on cause of death which is crucial for the calculation of CVD SMRs. Misclassification seems to be a problem [54,55], but there is no indication for systematic differences between ethnic groups. Cause of death information in our study was highly complete, which minimizes bias due to unascertained cause of death and allowed us to forego SMR correction. *Aussiedler *receive German citizenship upon entering the country, so they are included in the comparison data for the general population of Germany. This biases SMRs towards the null [56]. However, since *Aussiedler *from the FSU constitute less than two % of Germany's population, any resulting bias is negligible. We were confined to exclusively using data from population registries, so it was not possible to account for specific risk factors on an individual level, something which would have been highly desirable in the context of this population. Finally, we could only analyze mortality, and no objective and subjective morbidity measures.

## Conclusion

In contrast to what was expected, overall and CVD mortality among *Aussiedler *from the FSU is lower than in Germany's general population. There is no single stringent explanation, which underpins the fact that the reasons for health differences between migrants and the population of host countries, a widespread phenomenon, are still far from being sufficiently understood. We observed a secular rise in CVD SMRs, possibly because *Aussiedler *from the FSU do not benefit from improved CVD prevention and treatment. This, together with the fact that CVD SMRs are already high among younger *Aussiedler*, might lead to a considerable burden of CVD in this population in the future, which suggests that Public Health should pay more attention to this group. There is a definite need to assess possible reasons of shortcomings in CVD prevention for *Aussiedler*. The results of such assessment should help to improve future preventive measures, especially for younger *Aussiedler*. To be able to draw a clearer picture of their health status, further studies of quantitative as well as qualitative nature, including the assessment of individual risk factors, family structure and social networks, are required to learn more about health risks, but also health resources, in this and other migrant populations.

## Abbreviations

CVD cardiovascular disease

FSU former Soviet Union

ICD International Classification of Diseases

SES socio-economic status

SMR standardized mortality ratio

## Competing interests

The author(s) declare that they have no competing interests.

## Authors' contributions

HB and OR conceived of the study and the research questions. All authors participated in designing the study. UR and CK collected the data. UR and CK carried out the statistical analyses with the support of HB and OR. All authors interpreted the results, drafted and revised the manuscript and read and approved its final version.

## Pre-publication history

The pre-publication history for this paper can be accessed here:



## Figures and Tables

**Table 1:** Characteristics of all *Aussiedler *from the Former Soviet Union (FSU) to North-Rhine Westphalia (NRW) and of cohort members upon arrival in Germany together with the accrued person time in the respective categories of the covariables.

		**cohort**		**all *Aussiedler *from the FSU to NRW**
			
**covariable**	**category**	person years*	**individuals (%)**	**individuals (%)**
sex	male	117,597	16,734 (48.66)	133,807 (47.56)
	female	124,548	17,659 (51.34)	147,549 (52.44)
				
age group^§^	15–39	146,081	19,863 (57.75)	164,799 (58.57)
	40–64	75,180	10,954 (31.85)	88,467 (31.44)
	65+	20,884	3,576 (10.40)	28,090 (9.98)
				
duration of stay^†^	<5 years	146,891		
	5–8 years	83,130		
	>8 years	12,124		
				
study period^†^	1990–1993	26,494		
	1994–1997	78,096		
	1998–2002	137,555		
				
year of arrival	1990–1993	141,832	14,728 (42.82)	112,055 (39.83)
	1994–1997	76,404	11,441 (33.27)	104,275 (37.06)
	1998–2001	23,909	8,224 (23.91)	65,026 (23.11)
				
family size at arrival	single	41,092	5,512 (16.03)	44,264 (15.73)
	2–4	164,276	23,890 (69.46)	194,510 (69.13)
	>4	36,777	4,991 (14.51)	42,582 (15.13)
				
population size of first city of residence	<100,000	140,804	15,343 (44.61)	190,241 (67.62)
	> = 100,000	101,341	19,050 (55.39)	91,115 (32.38)
				
total		242,145	34,393(100.00)	281,356 (100.00)

*person time is rounded to full person years

^§^number of individuals and percentage are given for the age group at arrival in Germany

^†^since duration of stay and calendar period are time-dependent variables, the number of individuals and percentage in the categories cannot be given

**Table 2:** Observed and expected numbers of deaths (all causes) and standardised mortality ratios (SMR) in the whole cohort and the substrata during the follow-up period 1990–2001.

**covariable**	**category**	**observed deaths**	**expected deaths**	**SMR**	95%-CI*
whole cohort		1657	1960.09	**0.85**	0.81–0.89
					
Sex	male	870	976.59	**0.89**	0.83–0.95
	female	787	983.47	**0.81**	0.75–0.86
					
Age group	15–39	168	160.89	**1.04**	0.90–1.21
	40–64	562	613.67	**0.92**	0.84–0.99
	65+	927	1185.50	**0.78**	0.73–0.83
					
duration of stay	<5 years	958	1091.01	**0.88**	0.82–0.94
	5–8 years	594	747.43	**0.79**	0.73–0.86
	>8 years	105	121.62	**0.86**	0.71–1.05
					
study period	1990–1993	132	204.60	**0.65**	0.54–0.77
	1994–1997	519	629.08	**0.83**	0.76–0.90
	1998–2002	1006	1126.38	**0.89**	0.84–0.95
					
year of arrival	1990–1993	958	1247.33	**0.77**	0.72–0.82
	1994–1997	553	570.21	**0.97**	0.89–1.05
	1998–2001	146	142.51	**1.02**	0.87–1.20
					
family size at arrival	single	279	348.12	**0.80**	0.71–0.90
	2 to 4	1209	1343.33	**0.90**	0.85–0.95
	5+	169	268.60	**0.63**	0.54–0.73
					
size of first city of residence	>100,000	643	753.82	**0.85**	0.79–0.92
	<100,000	1014	1206.24	**0.84**	0.79–0.89

*95% confidence interval

**Table 3:** Observed and expected numbers of cardiovascular disease (CVD) deaths and standardised mortality ratios (SMR) in the whole cohort and the substrata during the follow-up period 1990–2001.

**covariable**	**category**	**observed CVD deaths**	**expected CVD deaths**	**SMR**	95%-CI*
whole cohort		680	865.59	**0.79**	0.73–0.85
					
Sex	male	296	389.09	**0.76**	0.68–0.85
	female	384	476.50	**0.81**	0.73–0.89
					
Age group	15–39	28	23.56	**1.19**	0.79–1.72
	40–64	165	199.91	**0.83**	0.71–0.96
	65+	487	642.12	**0.76**	0.69–0.83
					
duration of stay	<5 years	379	478.09	**0.79**	0.72–0.88
	5–8 years	250	333.67	**0.75**	0.66–0.85
	>8 years	51	53.82	**0.95**	0.72–1.25
					
study period	1990–1993	49	91.33	**0.54**	0.40–0.71
	1994–1997	228	281.17	**0.81**	0.71–0.92
	1998–2002	403	493.09	**0.82**	0.74–0.90
					
year of arrival	1990–1993	419	559.79	**0.75**	0.68–0.82
	1994–1997	215	247.67	**0.87**	0.76–0.99
	1998–2001	46	58.14	**0.79**	0.58–1.05
					
family size at arrival	single	125	154.97	**0.81**	0.68–0.96
	2 to 4	485	595.28	**0.81**	0.75–0.89
	5+	70	115.34	**0.61**	0.48–0.77
					
size of first city of residence	>100,000	403	537.33	**0.75**	0.68–0.83
	<100,000	277	327.58	**0.85**	0.75–0.95

*95% confidence intervals (CI) were calculated with approximate methods when the number of observed cases in a stratum was larger or equal than 50, and with exact methods if it was below.

**Table 4:** Rate ratios (RR) and 95% confidence intervals (CI) for all-cause standardised mortality ratios as derived from Poisson regression models which employ the natural logarithm of expected cases as offset term.

		**univariate**^†^		**multivariate-adjusted**^‡^	
**covariable**	**category**	**RR**	**95%-CI**	**RR**	**95%-CI**
Sex	male*	**1**		**1**	
	female	**0.90**	0.82–0.99	**0.94**	0.85–1.03
					
Age group	15–39*	**1**		**1**	
	40–64	**0.88**	0.74–1.04	**0.88**	0.74–1.04
	65+	**0.75**	0.64–0.99	**0.76**	0.64–0.90
					
duration of stay	<5 years*	**1**		**1**	
	5–8 years	**0.91**	0.82–1.00	**0.86**	0.74–1.00
	>8 years	**0.98**	0.80–1.20	**0.96**	0.75–1.23
					
study period	1990–1993*	**1**		**1**	
	1994–1997	**1.28**	1.06–1.55	**1.27**	1.04–1.55
	1998–2002	**1.38**	1.16–1.67	**1.44**	1.14–1.83
					
year of arrival	1990–1993*	**1**		**1**	
	1994–1997	**1.26**	1.13–1.40	**1.15**	1.01–1.31
	1998–2001	**1.33**	1.12–1.58	**1.11**	0.88–1.39
					
family size	single*	**1**		**1**	
	2 to 4	**1.12**	0.99–1.28	**1.09**	0.96–1.25
	5+	**0.79**	0.65–0.95	**0.76**	0.63–0.92
					
size of city	>100,000*	**1**		**1**	
	<100,000	**1.01**	0.92–1.12	**0.99**	0.90–1.10

*reference category

^†^the Poisson models only contain the respective covariable

^‡^the Poisson model contains all covariables; intercept (baseline log SMR): -0.24 (p = 0.05)

**Table 5:** Rate ratios (RR) and 95% confidence intervals (CI) for CVD standardised mortality ratios as derived from Poisson regression models which employ the natural logarithm of expected cases as offset term.

		**univariate**^†^		**multivariate-adjusted**^‡^	
**covariable**	**category**	**RR**	**95%-CI**	**RR**	**95%-CI**
Sex	male*	**1**		**1**	
	female	**1.06**	0.91–1.24	**1.10**	0.94–1.29
					
Age group	15–39*	**1**		**1**	
	40–64	**0.69**	0.47–1.06	**0.70**	0.47–1.06
	65+	**0.64**	0.44–0.96	**0.61**	0.43–0.92
					
duration of stay	<5 years*	**1**		**1**	
	5–8 years	**0.94**	0.80–1.11	**0.86**	0.68–1.08
	>8 years	**1.20**	0.88–1.58	**1.10**	0.76–1.59
					
study period	1990–1993*	**1**		**1**	
	1994–1997	**1.51**	1.12–2.08	**1.53**	1.12–2.13
	1998–2002	**1.52**	1.14–2.07	**1.65**	1.14–2.41
					
year of arrival	1990–1993*	**1**		**1**	
	1994–1997	**1.16**	0.98–1.36	**1.06**	0.87–1.29
	1998–2001	**1.06**	0.77–1.42	**0.89**	0.60–1.29
					
family size	single*	**1**		**1**	
	2 to 4	**1.01**	0.83–1.23	**1.00**	0.82–1.22
	5+	**0.75**	0.56–1.00	**0.74**	0.55–0.99
					
size of city	>100,000*	**1**		**1**	
	<100,000	**1.13**	0.97–1.31	**1.12**	0.96–1.30

*reference category

^†^the Poisson models only contain the respective covariable

^‡^the Poisson model contains all covariables; intercept (baseline log SMR): -0.25 (p = 0.31)
